# Associations of ω-3, ω-6 polyunsaturated fatty acids intake and ω-6: ω-3 ratio with systemic immune and inflammatory biomarkers: NHANES 1999-2020

**DOI:** 10.3389/fnut.2024.1410154

**Published:** 2024-06-07

**Authors:** Yifan Li, Hao Tang, Xiaotong Yang, Lili Ma, Hangqi Zhou, Guangjiang Zhang, Xin Chen, Lijun Ma, Jing Gao, Wei Ji

**Affiliations:** ^1^Department of Rheumatology, Affiliated Hospital of Nanjing University of Chinese Medicine, Nanjing, China; ^2^Department of Rheumatology, Liyang Hospital of Chinese Medicine, Liyang, China; ^3^Department of Rheumatology, Jiangsu Province Hospital of Chinese Medicine, Nanjing, China

**Keywords:** ω-3 PUFAs, ω-6 PUFAs, ω-6: ω-3 ratio, NHANES, systemic immune-inflammation index, neutrophil-to-lymphocyte ratio, platelet-lymphocyte ratio, white blood cell

## Abstract

**Background:**

In recent years, diseases caused by abnormal immune-inflammatory responses have become increasingly severe. Dietary intervention involving omega-3 polyunsaturated fatty acids (ω-3 PUFAs) has emerged as a potential treatment. However, research investigating the relationship between ω-3, ω-6 PUFAs, and ω-6 to ω-3 ratio with inflammatory biomarkers remains controversial.

**Methods:**

To investigate the correlation between the intake of ω-3 and ω-6 PUFAs and the ratio of ω-6: ω-3 with biomarkers of inflammation, the National Health and Nutrition Examination Survey (NHANES) data (1999 to 2020) was utilized. The systemic immune-inflammation index (SII), platelet-lymphocyte ratio (PLR), neutrophil-lymphocyte ratio (NLR), and white blood cell (WBC) were selected as study subjects. Dietary data for ω-3 and ω-6 PUFAs were collected via two 24-h dietary recall interviews. SII index and other indicators were obtained from the blood routine data. The multiple linear regression and restricted cubic spline models were utilized to evaluate the association of ω-3, ω-6 PUFAs intake, and ω-6: ω-3 ratio to SII and secondary measures.

**Results:**

This study involved a total of 43,155 American adults. ω-3 and ω-6 PUFAs exhibited negative correlations with SII, PLR, NLR, and WBC. The correlation between ω-6: ω-3 ratio and SII, PLR, NLR, and WBC was not significant. Furthermore, the dose–response relationship showed that the relationship between the intake of ω-3 and ω-6 PUFAs and SII was an “L” pattern.

**Conclusion:**

Intake of dietary ω-3 and ω-6 PUFAs reduces the levels of several inflammatory biomarkers in the body and exerts immunomodulatory effects.

## 1 Introduction

An immune-inflammatory response refers to the systemic response of the immune system of the body to a particular state. This response is involved not only in acute inflammation caused by infection or injury but also in the normal homeostatic regulation of the body (1, 2). However, prolonged chronic systemic inflammation elevates the risk of various disorders, including autoimmune disease, cardiovascular disease (CVD), cancer, and diabetes (3–7).

Dietary polyunsaturated fatty acids (PUFAs), serving as vital energy sources and cell membrane components, exert a crucial impact on human health (8, 9). Clinical trials and experimental research have demonstrated that ω-3 PUFAs possess significant anti-inflammatory properties (10, 11). Although ω-6 PUFAs are often theoretically considered pro-inflammatory mediators, the findings from clinical research do not consistently support this conventional hypothesis. Arachidonic acid (AA) supplementation was found to elevate AA content in human plasma or cellular phospholipids in a randomized controlled study and crossover design study conducted in the UK and US, respectively. However, it did not exert a significant impact on pro-inflammatory cytokine production and the number of inflammatory cells (12–14). Conversely, some investigations have even proposed that ω-6 PUFAs may be linked to decreased inflammation (15–17). Additionally, research on the relationship between ω-6: ω-3 ratio and inflammatory markers has yielded conflicting results. Numerous investigations have revealed that the proportion of ω-6: ω-3 is positively correlated with inflammatory markers (18–20). While Harris (21, 22) collated and analyzed 11 case–control and two prospective cohort studies, it was considered that the clinical ω-6: ω-3 ratio could not serve as a reliable indicator for predicting disease status or providing nutritional reference.

The concept of systemic immune-inflammation index (SII) was initially introduced by Hu (23) and has been applied in several disease areas, such as CVD, respiratory diseases, autoimmune diseases, and some cancers (4, 23–27). Additionally, platelet-lymphocyte ratio (PLR), neutrophil-lymphocyte ratio (NLR), and white blood cell (WBC) count are important indicators commonly used for early detection and prediction of inflammatory diseases in clinical practice and have also been widely used in clinical studies (28–30).

Clinical interventions and experimental studies on dietary ω-3 and ω-6 PUFAs have not elucidated the relationship between the two and inflammatory mediators. Similarly, there is no consensus on the effects of the optimal ratio of ω-6 to ω:3 PUFA in humans. Therefore, this study investigated a dataset of ethnically diverse cohorts of Americans aged 20 years and older from the National Health and Nutrition Examination Survey (NHANES) data to analyze the relationship between intake of ω-3, ω-6 PUFAs and the ratio of ω-6 to ω:3 and systemic immune-inflammatory markers to provide more compelling evidence for clinical interventions and therapies.

## 2 Materials and methods

### 2.1 Study population

**Figure 1:**
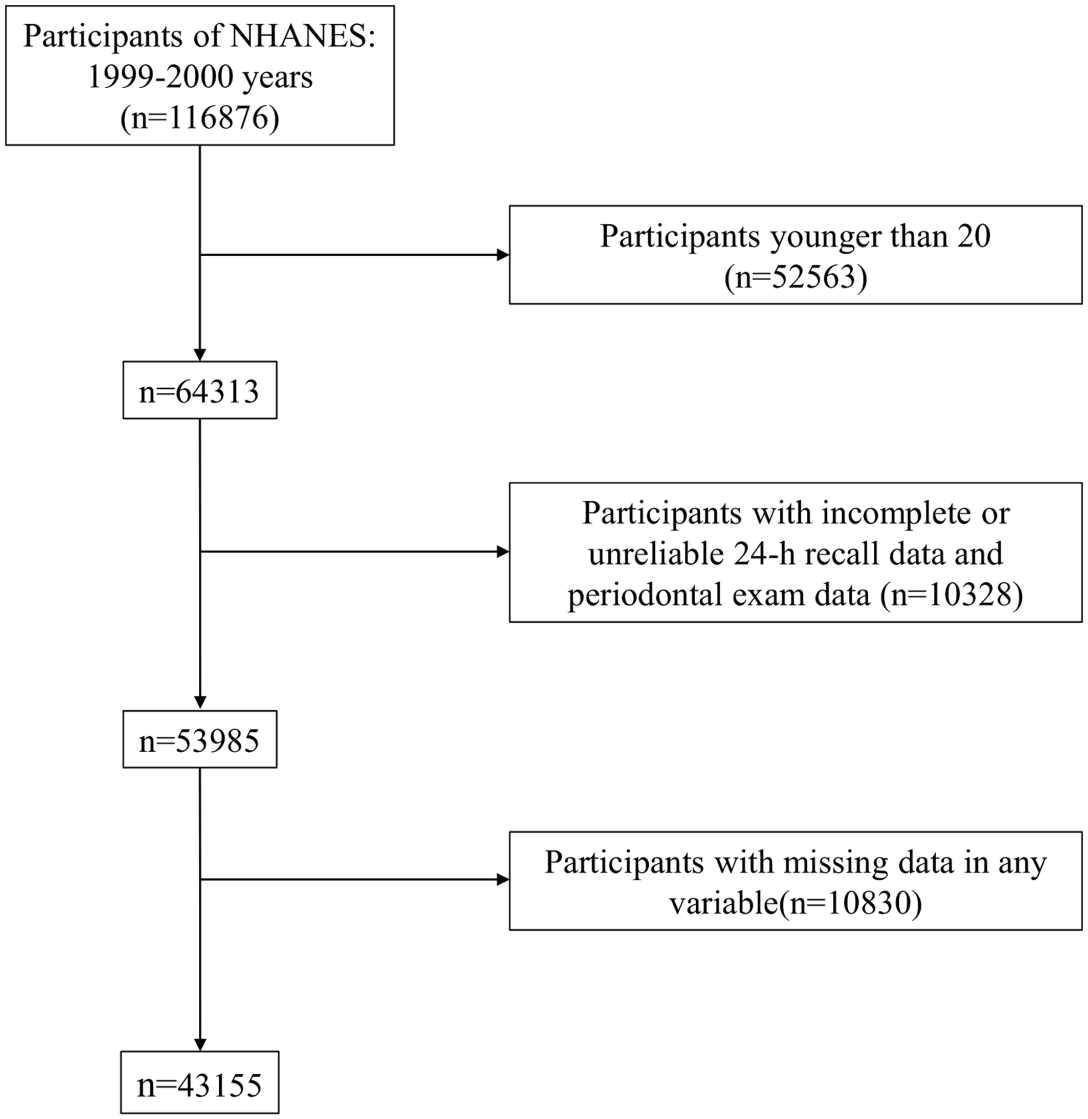
Flowchart of the screening process for the selection of eligible participants.

The National Health and Nutrition Examination Survey (NHANES), carried out by the Centers for Disease Control and Prevention (CDC), is a cross-sectional survey undertaken on a biennial basis. Its purpose is to analyze the nutritional and health status of children and adults in the US. This assessment is carried out by selecting a representative sample of the US population employing a complex multistage probability sampling design (31). NHANES contains interviews covering demographic, dietary, health-related, and socio-economic issues, alongside laboratory tests performed by highly qualified medical personnel (32). For this study, we included 116,876 participants who took part in the survey during the period 1999–2020. Our study exclusion criteria were as follows: (1) adults younger than 20 years of age (*n* = 52,563) (2) dietary fatty acid data were incomplete or abnormal and missing laboratory tests (*n* = 10,328) (3) any other covariates were missing (*n* = 10,830). After that, this study comprised 43,155 individuals, including 22,575 women and 20,580 men (age: ≥20 years) (Figure 1).

### 2.2 ω-3 and ω-6 PUFAs dietary intake

During the Mobile Examination Centre (MEC) portion of NHANES, ω-3 and ω-6 PUFAs dietary intake was obtained through two 24-h dietary recalls administered 3 to 10 days apart. The main diet interview was conducted in the MEC, and subsequent diet interview data were obtained by the Home Office via telephone. Comprehensive descriptions of the data processing procedures and diet interviews are available in the Diet Interview section of the NHANES website. α-linoleic acid (ALA, 18:3), docosapentaenoic acid (DPA, 22:5), docosahexaenoic acid (DHA, 22:6), eicosapentaenoic acid (EPA, 20:5), and stearidonic acid (SDA, 18:4) are constituents of ω-3 PUFAs. On the other hand, AA (20:4) and linoleic acid (LA, 18:2) are included in ω-6 PUFAs. Dietary intake of ω-3, ω-6 PUFAs, and ω-6: ω-3 ratios were categorized into tertiles for subsequent analyses.

### 2.3 SII and secondary test results

The primary outcome was SII calculated as platelet count × neutrophil count/lymphocyte count (33). NLR = Neutrophil count/lymphocyte count. PLR = platelet count/lymphocyte count. WBC count was obtained directly from a blood routine. NHANES implemented standardized protocols for the measurement of these biomarkers, and all investigators obtained written informed consent from participants.

### 2.4 Selection of covariates

Alongside the investigation of ω-3 and ω-6 PUFAs dietary intake, several potential confounders were examined, including age (20–40, 41–60, and ≥ 60 years), body mass index (BMI) (normal: <25 kg/m^2^; overweight: 25–30 kg/m^2^; obesity: ≥30 kg/m^2^), educational level (below high school, high school, or above), marital status (married/cohabiting with partner or divorced/widowed/never married/separated), poverty income ratio (PIR) (below poverty line: ≤0.99, above poverty line: ≥1), race (Mexican Americans, non-Hispanic whites, non-Hispanic blacks, other Hispanics, and other races), smoking status (never, before, and now), and sex (men and women).

### 2.5 Statistical analyses

Due to the complex sampling design employed by NHANES, all experiments were adjusted for weighted variables and survey design to ensure that the included population was nationally representative. The Kolmogorov–Smirnov normality test was utilized to examine the normality of continuous variables, which are expressed as mean ± standard error. Variables that were not normally distributed were presented utilizing the median (interquartile range). Adjusted dietary intake of ω-3 and ω-6 PUFAs was categorized into three groups based on tertiles, with the lowest tertile serving as the reference value. Multivariate weighted linear regression models were utilized to determine the correlation between dietary ω-3, ω-6 PUFAs, and ω-6: ω-3 ratios and SII as well as other secondary outcomes. For each regression analysis, a total of three statistical models were developed. Model 1 remained unadjusted, while Model 2 was adjusted for age and sex only. Model 3 encompassed adjustments for all covariates, including age, BMI, education, monthly household poverty level index, marital status, race, sex, and smoking status. Fully adjusted models considered demographic, dietary, lifestyle, and metabolic factors. To further investigate the dose–response correlation between dietary ω-3 and ω-6 PUFAs, as well as the ω-6: ω-3 ratios with the primary measure SII, the restricted cubic splines were applied. These splines included three nodes at the 5th, 50th, and 95th percentiles of the exposure distribution in multivariate-adjusted model 3. All statistical analyses were performed using R software for data analysis. All statistical tests were two-sided, and *p* < 0.05 was deemed to reflect statistical significance.

## 3 Results

### 3.1 Baseline attributes

Tables 1–3 present the baseline attributes of the study population, categorized by dietary intake of ω-3, ω-6 PUFAs, and ω-6: ω-3 ratios in triple-digit groups, respectively. The study comprised 43,155 participants in total. The tertile intervals for ω-3 fatty acid intake were: low intake (≤ 1.167 g/day, *n* = 14,401), medium intake (1.167–1.928 g/day, *n* = 14,364), and high intake (>1.928 g/day, *n* = 14,390). Tertile intervals for ω-6 fatty acid intake were defined as low dose intake (≤ 10.929 g/day, *n* = 14,387), medium dose intake (10.929–17.63 g/day, *n* = 14,384), and high dose intake (>17.63 g/day, *n* = 14,384). The tertile interval for the ω-6: ω-3 proportion of fatty acid intake was: low (≤ 8.19, *n* = 14,383), medium (8.19–10.13, *n* = 14,383), and high (≥10.13, *n* = 14,389). Participants with higher ω-3 and ω-6 PUFAs intake tended to be young and medium-aged, male, married or cohabiting with a partner, non-Hispanic white, higher educational level, wealthier, and non-smoking. Regarding laboratory parameters, individuals with higher ω-3 and ω-6 PUFAs intake demonstrated lower levels of SII, PLR, and WBC.

**Table 1:** Weighted characteristics of the study population based on dietary ω-3 fatty acids intake.

	Total ω-3 fatty acids intake (g)			*p*-value
Variable	<=1.167 (*n* = 14,401)	1.167–1.928 (*n* = 14,364)	>1.928 (*n* = 14,390)	
Age group (%)				**< 0.0001**
20–40	4,713 (38.0)	5,147 (40.4)	5,328 (39.4)	
41–60	4,440 (36.0)	4,760 (35.6)	5,065 (38.5)	
> = 60	5,248 (26.0)	4,457 (24.0)	3,997 (22.0)	
Sex (%)				**< 0.0001**
Female	8,946 (63.6)	7,592 (53.6)	6,037 (40.9)	
Male	5,455 (36.4)	6,772 (46.4)	8,353 (59.1)	
Marital status (%)				**< 0.0001**
Married/Living with partner	8,494 (60.9)	8,900 (64.3)	9,162 (66.7)	
Windowed/Divorced/Separated/Never married	5,907 (39.1)	5,464 (35.7)	5,228 (33.3)	
Race (%)				**0.01**
Mexican American	2,723 (8.6)	2,277 (8.0)	2037 (7.5)	
Non-Hispanic White	6,492 (68.4)	6,743 (69.6)	6,761 (70.4)	
Non-Hispanic Black	2,899 (10.9)	2,891 (10.4)	3,086 (10.0)	
Other Hispanic	1,186 (5.7)	1,177 (5.2)	1,039 (5.0)	
Other race	1,101 (6.3)	1,276 (6.8)	1,467 (7.1)	
Education level (%)				**< 0.0001**
Below high school	2048 (6.8)	1,287 (4.4)	900 (3.3)	
High school	5,742 (39.0)	5,346 (34.0)	4,802 (30.4)	
Above high school	6,611 (54.1)	7,731 (61.7)	8,688 (66.3)	
Smoking status (%)				**< 0.0001**
never	7,842 (54.0)	7,948 (55.6)	7,764 (54.5)	
former	3,528 (22.6)	3,628 (24.9)	3,920 (27.7)	
current	3,031 (23.4)	2,788 (19.5)	2,706 (17.8)	
Body mass index (%)				**0.003**
<25	4,115 (31.7)	4,046 (30.6)	4,041 (28.5)	
25–30	4,810 (32.1)	4,931 (33.3)	4,824 (34.5)	
>30	5,476 (36.2)	5,387 (36.1)	5,525 (37.0)	
Poverty income ratio (%)				**< 0.0001**
<=0.99	3,241 (16.8)	2,670 (12.9)	2,304 (10.8)	
> = 1	11,160 (83.2)	11,694 (87.1)	12,086 (89.2)	
SII	578.4 (4.3)	552.5 (4.5)	543.7 (4.4)	**< 0.0001**
NLR	2.2 (0.0)	2.2 (0.0)	2.2 (0.0)	0.1
PLR	131.1 (0.7)	127.4 (0.7)	127.7 (0.7)	**< 0.0001**
WBC (x109)	7.4 (0.0)	7.3 (0.0)	7.3 (0.0)	**0.002**

SII, systemic immune-inflammation index; NLR, neutrophil-to-lymphocyte ratio; PLR, platelet-lymphocyte ratio; WBC: white blood cell.

Bold values represent statistical significance.

**Table 2:** Weighted characteristics of the study population based on dietary ω-6 fatty acids intake.

	Total ω-6 fatty acids intake (g)			*p-*value
Variable	<=10.929 (*n* = 14,387)	10.929–17.63 (*n* = 14,384)	>17.63 (*n* = 14,384)	
Age group (%)				**< 0.0001**
20–40	4,464 (35.8)	5,081 (39.9)	5,643 (41.7)	
41–60	4,358 (35.7)	4,761 (35.8)	5,146 (38.6)	
> = 60	5,565 (28.5)	4,542 (24.3)	3,595 (19.7)	
Sex (%)				**< 0.0001**
Female	9,073 (65.3)	7,750 (55.3)	5,752 (38.3)	
Male	5,314 (34.7)	6,634 (44.7)	8,632 (61.7)	
Marital status (%)				**< 0.0001**
Married/Living with partner	8,521 (61.6)	8,978 (64.6)	9,057 (65.7)	
Windowed/Divorced/Separated/Never married	5,866 (38.4)	5,406 (35.4)	5,327 (34.3)	
Race (%)				**< 0.0001**
Mexican American	2,629 (8.4)	2,347 (8.2)	2061 (7.6)	
Non-Hispanic White	6,367 (67.4)	6,741 (70.0)	6,888 (70.9)	
Non-Hispanic Black	2,765 (10.4)	6,741 (70.1)	3,271 (10.8)	
Other Hispanic	1,380 (6.6)	6,741 (70.2)	920 (4.6)	
Other race	1,246 (7.2)	6,741 (70.3)	1,244 (6.2)	
Education level (%)				**< 0.0001**
Below high school	2,153 (7.6)	1,286 (4.2)	796 (2.9)	
High school	5,655 (38.5)	5,320 (33.5)	4,915 (31.4)	
Above high school	6,579 (53.9)	7,778 (62.3)	8,673 (65.7)	
Smoking status (%)				**< 0.0001**
never	8,008 (54.0)	7,971 (56.6)	7,575 (53.6)	
former	3,517 (23.5)	3,663 (24.1)	3,896 (27.6)	
current	2,862 (22.5)	2,750 (19.3)	2,913 (18.8)	
Body mass index (%)				**< 0.001**
<25	4,133 (31.7)	4,146 (31.0)	3,923 (28.2)	
25–30	4,921 (32.5)	4,876 (33.7)	4,768 (33.8)	
>30	5,333 (35.9)	5,362 (35.3)	5,693 (38.0)	
Poverty income ratio (%)				**< 0.0001**
<=0.99	3,260 (16.8)	2,607 (12.5)	2,348 (11.2)	
> = 1	11,127 (83.2)	11,777 (87.5)	12,036 (88.8)	
SII	576.6 (4.5)	552.9 (4.2)	545.4 (4.2)	**< 0.0001**
NLR	2.2 (0.0)	2.2 (0.0)	2.2 (0.0)	0.1
PLR	131.0 (0.7)	127.9 (0.7)	127.4 (0.7)	**< 0.001**
WBC (x109)	7.4 (0.0)	7.4 (0.0)	7.3 (0.0)	0.01

SII, systemic immune-inflammation index; NLR, neutrophil-to-lymphocyte ratio; PLR, platelet-lymphocyte ratio; WBC: white blood cell.

Bold values represent statistical significance.

**Table 3:** Weighted characteristics of the study population based on dietary ω-6: ω-3 ratio.

	ω-6: ω-3 ratio			*p*-value
Variable	<=8.19 (*n* = 14,383)	8.19–10.13 (*n* = 14,383)	>10.13 (*n* = 14,389)	
Age group (%)				**< 0.0001**
20–40	4,495 (35.6)	5,196 (40.0)	5,497 (42.1)	
41–60	4,688 (36.8)	4,708 (36.0)	4,869 (37.5)	
> = 60	5,200 (27.5)	4,479 (23.9)	4,023 (20.5)	
Sex (%)				**< 0.0001**
Female	7,848 (55.7)	7,514 (51.1)	7,213 (50.0)	
Male	6,535 (44.3)	6,869 (48.9)	7,176 (50.0)	
Marital status (%)				0.2
Married/Living with partner	8,981 (64.6)	8,789 (64.4)	8,786 (63.3)	
Windowed/Divorced/Separated/Never married	5,402 (35.4)	5,594 (35.6)	5,603 (36.7)	
Race (%)				**< 0.0001**
Mexican American	2,116 (7.4)	2,449 (8.4)	2,472 (8.2)	
Non-Hispanic White	6,395 (67.9)	6,833 (70.2)	6,768 (70.4)	
Non-Hispanic Black	2,688 (9.3)	2,869 (10.0)	3,319 (11.8)	
Other Hispanic	1,492 (6.7)	1,128 (5.5)	782 (3.8)	
Other race	1,692 (8.7)	1,104 (5.9)	1,048 (5.8)	
Education level (%)				**< 0.0001**
Below high school	1,562 (5.6)	1,359 (4.5)	1,314 (4.2)	
High school	4,939 (31.2)	5,420 (35.6)	5,531 (35.8)	
Above high school	7,882 (63.2)	7,604 (59.9)	7,544 (59.9)	
Smoking status (%)				**< 0.0001**
never	8,168 (55.9)	7,863 (55.2)	7,523 (53.2)	
former	3,729 (26.0)	3,615 (24.3)	3,732 (25.2)	
current	2,486 (18.1)	2,905 (20.5)	3,134 (21.5)	
Body mass index (%)				**< 0.0001**
<25	4,308 (31.8)	3,872 (28.3)	4,022 (30.5)	
25–30	5,021 (35.0)	4,736 (32.5)	4,808 (32.6)	
>30	5,054 (33.2)	5,775 (39.1)	5,559 (36.9)	
Poverty income ratio (%)				0.5
<=0.99	2,714 (13.2)	2,699 (13.1)	2,802 (13.7)	
> = 1	11,669 (86.8)	11,684 (86.9)	11,587 (86.3)	
SII	555.5 (4.6)	564.2 (4.9)	552.5 (3.9)	0.1
NLR	2.2 (0.0)	2.2 (0.0)	2.2 (0.0)	**0.01**
PLR	128.6 (0.7)	128.8 (0.7)	128.5 (0.7)	1
WBC (x109)	7.3 (0.0)	7.4 (0.0)	7.3 (0.0)	0.2

SII, systemic immune-inflammation index; NLR, neutrophil-to-lymphocyte ratio; PLR, platelet-lymphocyte ratio; WBC: white blood cell.

Bold values represent statistical significance.

### 3.2 Associations between dietary ω-3, ω-6 PUFAs intake and ω-6: ω-3 ratio and SII, PLR, NLR, WBC

Table 4 shows the relationship between the dietary intake of ω-3, ω-6 PUFAs and ω-6: ω-3 ratio and SII. In all three models, there was a clear negative correlation between ω-3 and ω-6 PUFAs intake and SII. In model 1, the effect size (β) and 95% confidence intervals (CI) for SII were −34.662 (−46.069, −23.256) and − 31.157 (−41.912, −20.402) for the high-dose intake group of ω-3 and ω-6 PUFAs, respectively. In model 2, there was a negative relationship between the high-dose intake group of ω-3 and ω-6 PUFAs and SII, with *β* and 95% CI of −25.004 (−36.653, −13.354) and −18.021 (−29.131, −6.911), respectively. In model 3, a negative relationship was found between the high-dose intake group of ω-3 and ω-6 PUFAs and SII, with β and 95% CI of −21.309 (−33.098, −9.520) and −15.557 (−26.681, −4.434), respectively. The *p*-values for trend were statistically significant for ω-3 and ω-6 PUFAs intake (*p* trend <0.001). However, the correlation between the proportion of ω-6: ω-3 fatty acid intake and SII was not statistically significant. In Model 2, there was a positive correlation between the medium scale group of ω-6: ω-3 ratios and SII, with *β* and 95% CI of 12.163 (0.127, 24.199).

**Table 4:** Survey-weighted multivariate regression analyses of associations between dietary ω-3 and ω-6 PUFAs intake and ω-6:ω-3 ratio and SII.

SII	Model 1 *β* (95%CI) *p*-value	Model 2 *β* (95%CI) *p*-value	Model 3 *β* (95%CI) *p*-value
Total ω-3 PUFAs intake (g)			
<=1.167	ref	ref	ref
1.167–1.928	−25.9 (−36.554, −15.246)**	−21.198 (−32.102, −10.295)**	−18.628 (−29.451, −7.806)**
>1.928	−34.662 (−46.069, −23.256)**	−25.004 (−36.653, −13.354)**	−21.309 (−33.098, −9.520)**
*p* for trend	<0.0001	<0.0001	<0.001
Total ω-6 PUFAs intake (g)			
<=10.929	ref	ref	ref
10.929–17.63	−23.686 (−34.169, −13.203)**	−18.418 (−28.841, −7.996)**	−16.115 (−26.451, −5.780)**
>17.63	−31.157 (−41.912, −20.402)**	−18.021 (−29.131, −6.911)**	−15.557 (−26.681, −4.434)**
*p* for trend	<0.0001	<0.0001	<0.001
Total ω-6:ω-3 ratio			
<=8.19	ref	ref	ref
8.19–10.13	8.65 (−3.452, 20.752)	12.163 (0.127, 24.199)*	8.198 (−3.488, 19.884)
>10.13	−3.061 (−13.079, 6.956)	1.943 (−8.029, 11.914)	−0.583 (−10.800, 9.633)
*p* for trend	0.524	0.732	0.867

SII, systemic immune-inflammation index; *β*, standardized coefficients; CI, confidence interval. Non-adjusted model: no covariates were adjusted. Minimally-adjusted model: age and gender were adjusted. Fully-adjusted model: age, gender, race, marital status, education level, smoking status, body mass index, and family monthly poverty level index were adjusted. *p < 0.05; **p < 0.01.

Table 5 shows the dietary intake of ω-3, ω-6 PUFAs, and the relationship between ω-6: ω-3 ratio and PLR. In model 1, the *β* and 95% CI for PLR were −3.369 (−5.145, −1.593) and −3.606 (−5.428, −1.784) for the high-dose intake group of ω-3 and ω-6 PUFAs, respectively. In model 2, there was an inverse relationship between the high-dose intake group of ω-3 and ω-6 PUFAs and PLR, with *β* and 95% CI of −2.777 (−4.595, −0.959) and −1.958 (−3.608, −0.308), respectively. In model 3, a negative relationship was found between the high-dose intake group of ω-3 and ω-6 PUFAs and PLR, with *β* and 95% CI of −2.555 (−4.374, −0.735) and −1.867 (−3.702, −0.033), respectively. The *p-*values for trend for ω-3 and ω-6 PUFAs intake were statistically significant (*p* < 0.001). The correlation between ω-6: ω-3 ratio and PLR did not exhibit statistical significance.

**Table 5:** Survey-weighted multivariate regression analyses of associations between dietary ω-3 and ω-6 PUFAs intake and ω-6:ω-3 ratio and PLR.

PLR	Model 1 *β* (95%CI) *p*-value	Model 2 *β* (95%CI) *p*-value	Model 3 *β* (95%CI) *p*-value
Total ω−3 PUFAs intake (g)			
<=1.167	ref	ref	ref
1.167–1.928	−3.731 (−5.511, −1.951)**	−2.777 (−4.595, −0.959)**	−3.499 (−5.292, −1.706)**
>1.928	−3.369 (−5.145, −1.593)**	−1.576 (−3.365, 0.213)	−2.555 (−4.374, −0.735)**
*p* for trend	<0.001	0.1	<0.01
Total ω−6 PUFAs intake (g)			
<=10.929	ref	ref	ref
10.929–17.63	−3.128 (−4.802, −1.455)**	−1.958 (−3.608, −0.308)**	−2.782 (−4.395, −1.169)**
>17.63	−3.606 (−5.428, −1.784)**	−0.882 (−2.711, 0.947)	−1.867 (−3.702, −0.033)*
*p* for trend	<0.001	0.383	0.059
Total ω−6:ω−3 ratio			
<=8.19	ref	ref	ref
8.19–10.13	0.161 (−1.679, 2.002)	1.053 (−0.765, 2.872)	1.494 (−0.190, 3.177)
>10.13	−0.117 (−1.891, 1.656)	1.233 (−0.512, 2.979)	1.574 (−0.203, 3.352)
*p* for trend	0.893	0.168	0.086

PLR, platelet-lymphocyte ratio; *β*, standardized coefficients; CI, confidence interval. Non-adjusted model: no covariates were adjusted. Minimally-adjusted model: age and gender were adjusted. Fully-adjusted model: age, gender, race, marital status, education level, smoking status, body mass index, and family monthly poverty level index were adjusted. *p < 0.05; **p < 0.01.

Table 6 depicts the dietary intake of ω-3, ω-6 PUFAs, and the relationship between ω-6: ω-3 ratio and NLR. In model 1, the *β* and 95% CI for NLR were −0.041 (−0.076, −0.006) and −0.04 (−0.077, −0.003) for the medium-dose intake group of ω-3 and ω-6 PUFAs, respectively. In model 2, there was an inverse relationship between the high-dose intake group of ω-3 and ω-6 PUFAs and NLR, with *β* and 95% CI of −0.045 (−0.084, −0.005) and −0.038 (−0.075, −0.001), respectively. In model 3, a negative relationship was found between the medium-dose intake group of ω-3 PUFAs and NLR, with *β* and 95% CI of −0.037 (−0.071, −0.002). The *p-*values for trend for ω-3 and ω-6 PUFAs intake were statistically significant (*p* < 0.05). In Model 1, there was a negative correlation between the high-scale group of ω-6: ω-3 ratios and NLR, with *β* and 95% CI of −0.037 (−0.073, −0.002).

**Table 6:** Survey-weighted multivariate regression analyses of associations between dietary ω-3 and ω-6 PUFAs intake and ω-6:ω-3 ratio and NLR.

NLR	Model 1 *β* (95%CI) *p*-value	Model 2 *β* (95%CI) *p*-value	Model 3 *β* (95%CI) *p*-value
Total ω−3 PUFAs intake (g)			
<=1.167	ref	ref	ref
1.167–1.928	−0.041 (−0.076, −0.006)*	−0.042 (−0.077, −0.007)*	−0.037 (−0.071, −0.002)*
>1.928	−0.032 (−0.072, 0.008)	−0.045 (−0.084, −0.005)*	−0.036 (−0.076, 0.004)
*p* for trend	0.188	<0.05	0.082
Total ω−6 PUFAs intake (g)			
<=10.929	ref	ref	ref
10.929–17.63	−0.04 (−0.077, −0.003)*	−0.034 (−0.070, 0.002)	−0.031 (−0.067, 0.005)
>17.63	−0.039 (−0.077, −0.001)*	−0.038 (−0.075, −0.001)*	−0.033 (−0.070, 0.004)
*p* for trend	<0.05	<0.05	0.086
Total ω−6:ω−3 ratio			
<=8.19	ref	ref	ref
8.19–10.13	0.02 (−0.022, 0.063)	0.033 (−0.009, 0.075)	0.024 (−0.017, 0.065)
>10.13	−0.037 (−0.073, −0.002)*	−0.014 (−0.050, 0.021)	−0.02 (−0.055, 0.016)
*p* for trend	<0.05	0.402	0.263

NLR, neutrophil-to-lymphocyte ratio; β, standardized coefficients; CI, confidence interval. Non-adjusted model: no covariates were adjusted. Minimally-adjusted model: age and gender were adjusted. Fully-adjusted model: age, gender, race, marital status, education level, smoking status, body mass index, and family monthly poverty level index were adjusted. *p < 0.05; **p < 0.01.

Table 7 shows the ω-3 and ω-6 PUFAs dietary intake and the relationship between ω-6: ω-3 ratio and WBC. In model 1, the *β* and 95% CI for WBC were −0.169 (−0.264, −0.073) and − 0.128 (−0.214, −0.041) for the high-dose intake group of ω-3 and ω-6 PUFAs, respectively. The *p*-values trend for ω-3 and ω-6 PUFAs intake were less than 0.01 and 0.05, respectively, and were statistically significant. In model 2, there was a negative relationship between the high-dose intake group of ω-3 and ω-6 PUFAs and WBC, with *β* and 95% CI of −0.142 (−0.246, −0.039) and-0.113 (−0.206, −0.021), respectively. The *p*-value for trend for ω-3 PUFAs intake was statistically significant. (*p* < 0.01). No notable relationship was depicted between ω-6: ω-3 ratio and WBC.

**Table 7:** Survey-weighted multivariate regression analyses of associations between dietary ω-3 and ω-6 PUFAs intake and ω-6:ω-3 ratio and WBC.

WBC	Model 1 *β* (95%CI) *p*-value	Model 2 *β* (95%CI) *p*-value	Model 3 *β* (95%CI) *p*-value
Total ω−3 PUFAs intake (g)			
<=1.167	ref	ref	ref
1.167–1.928	−0.098 (−0.175, −0.021)*	−0.091 (−0.169, −0.014)*	−0.023 (−0.098, −0.051)
>1.928	−0.169 (−0.264, −0.073)**	−0.142 (−0.246, −0.039)**	−0.049 (−0.148, 0.050)
*p* for trend	<0.01	<0.01	0.334
Total ω−6 PUFAs intake (g)			
<=10.929	ref	ref	ref
10.929–17.63	−0.043 (−0.139, 0.054)	−0.042 (−0.142, 0.058)	0.028 (−0.067, 0.123)
>17.63	−0.128 (−0.214, −0.041)**	−0.113 (−0.206, −0.021)*	−0.037 (−0.121, 0.048)
*p* for trend	<0.05	0.015	0.354
Total ω−6:ω−3 ratio			
<=8.19	ref	ref	ref
8.19–10.13	0.06 (−0.031, 0.151)	0.051 (−0.038, 0.140)	−0.024 (−0.109, 0.060)
>10.13	−0.006 (−0.108, 0.097)	−0.024 (−0.124, 0.076)	−0.074 (−0.171, 0.023)
*p* for trend	0.899	0.625	0.132

WBC, white blood cell; *β*, standardized coefficients; CI, confidence interval. Non-adjusted model: no covariates were adjusted. Minimally-adjusted model: age and gender were adjusted. Fully-adjusted model: age, gender, race, marital status, education level, smoking status, body mass index, and family monthly poverty level index were adjusted. *p < 0.05; **p < 0.01.

### 3.3 Stratified analyses of the associations between dietary ω-3, ω-6 PUFAs intake and SII

The study participants were divided into groups based on sex, age, smoking status, race, BMI, and education (Supplementary Tables S1, S2). The analysis showed that the relationship between dietary intake of ω-3 and ω-6 fatty acids and the SII index remained consistent across all the groups and did not exhibit significant variations. However, it is important to note that the association between ω-3 and ω-6 fatty acid intake and SII was found to be stronger in the obese population (BMI > 30).

### 3.4 Nonlinear associations between dietary ω-3, ω-6 PUFAs intake and SII

**Figure 2:**
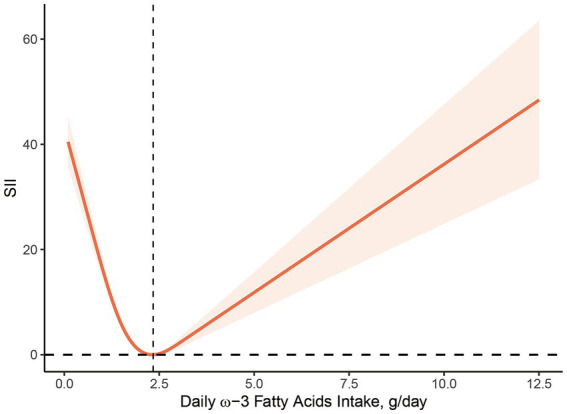
Dose–response relationship between ω-3 PUFAs intake and SII. The association was adjusted for age, gender, race, marital status, education level, smoking status, body mass index, family monthly poverty level index.

**Figure 3:**
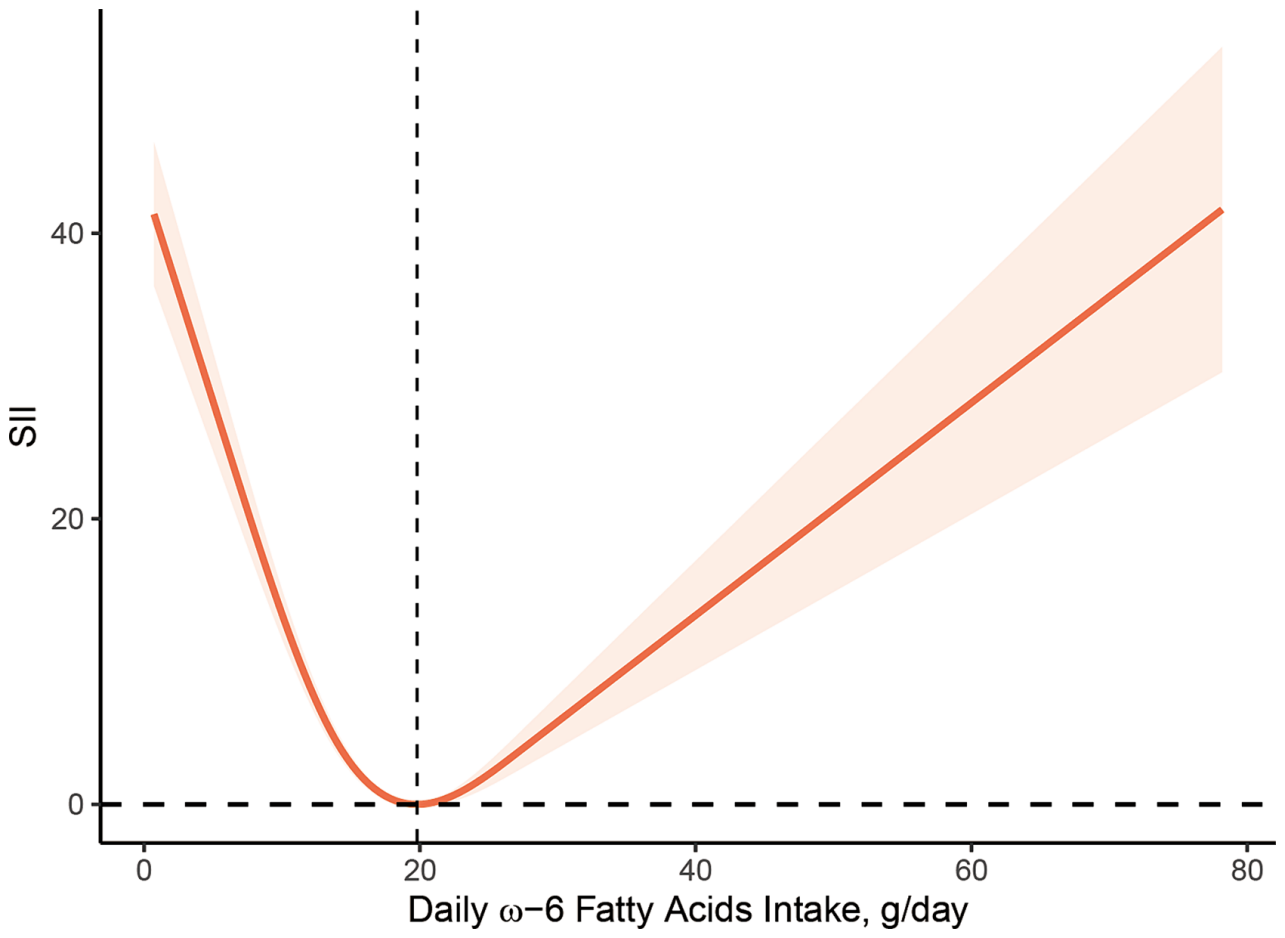
Dose–response relationship between ω-6 PUFAs intake and SII. The association was adjusted for age, gender, race, marital status, education level, smoking status, body mass index, family monthly poverty level index.

Figures 2, 3 illustrate the findings of the dose–response relationship between ω-3, ω-6 PUFAs and SII index. As shown in the figure, an L-type correlation was observed between ω-3 and ω-6 PUFAs intake and SII (*p* for nonlinearity <0.05). The dose–response relationship between intake of ω-3 and ω-6 PUFAs and SII showed an overall trend of first decrease and then increase, with inflection points of 2.35 g/day and 19.79 g/day, respectively. However, the non-linear relationship between the ω-3: ω-6 ratio and SII was not significant (*p* for nonlinearity >0.05).

## 4 Discussion

A comprehensive cross-sectional survey was carried out in this research to assess the relationship between dietary intake of ω-3, ω-6 PUFAs, and ω-6 to ω-3 ratio with systemic immune and inflammatory biomarkers. Data from the NHANES survey for 1999 to 2020, representing the US population, were utilized. In this study, we found a significant negative correlation between dietary intake of ω-3 and ω-6 PUFAs and SII, NLR, PLR, and WBC, which supports the contention that ω-3 PUFAs exert an anti-inflammatory effect, and that ω-6 PUFAs have a similar anti-inflammatory effect. In addition, the dose–response relationship suggests that ω-3 and ω-6 PUFAs intakes are associated with SII in a non-linear L-form, whereas ω-6: ω-3 ratios are not substantially related to these inflammatory biomarkers.

Theoretical studies suggest that ω-3 and ω-6 PUFAs compete with each other at cyclooxygenase (COX) and lipoxygenase (LOX) sites to generate different types of eicosanoids, such as prostaglandins (PGs) and leukotrienes (LTs), etc. (34). In response to inflammatory stimuli, AA within the ω-6 PUFA is released from membrane phospholipids. Subsequently, it undergoes conversion into PGE2 and LTB4 in a COX- and LOX-dependent manner. This process exerts a strong pro-inflammatory effect, contributing to platelet aggregation and vasoconstriction. In contrast, the metabolism of EPA and DHA in ω-3 PUFA produces PGE3 and LTB5, which exhibit anti-inflammatory and antiplatelet aggregation effects (35).

However, the relationship between fatty acid elongation and desaturase action is rather complex, and clinical outcome is not easily predicted based on biochemical pathways alone. The association between dietary PUFAs and inflammatory markers has not yet been fully elucidated (36). The anti-inflammatory impacts of ω-3 fatty acids have been extensively documented in multiple clinical and experimental studies. Two extensive studies by Derosa et al. (10, 11) revealed substantially reduced levels of serum of high-sensitivity C-reactive protein (hs-CRP), matrix metalloproteinase (MMP)-2, and MMP-9 in patients with dyslipidemia after 6 months of EPA + DHA intake at 2.6 g/day compared with placebo. ω-3 PUFAs also protect from CVD by addressing arrhythmias, lowering blood pressure, plasma homocysteine and serum triglycerides, prolonging clotting time, and suppressing platelet aggregation (37). This is consistent with the findings of this research. However, it is crucial to highlight that there is no substantial correlation between consuming low doses of ω-3 fatty acids over short courses and inflammation biomarkers (38).

At the same time, it was found that ω-6 PUFAs also had some anti-inflammatory effects. Although ω-6 PUFAs are theoretically and widely recognized as pro-inflammatory mediators, multiple clinical studies have failed to substantiate this hypothesis (12–14). In a study examining the impact of dietary AA supplementation on peripheral blood mononuclear cells (PBMCs), participants who received 0.7 g/day of AA-rich single-cell oil (ARASCO) for 12 weeks exhibited an elevation in the proportion of AA in PBMC phospholipids of total fatty acids (12). In another study, ARASCO supplementation for 7 weeks significantly increased the secretion of PGE2 and LTB4 from lipopolysaccharide-stimulated cultured PBMCs. However, this supplementation did not lead to the increased secretion of interleukin-6 (IL-6), IL-1β, or tumor necrosis factor-α (TNF-α), nor did it affect the number of circulating lymphocytes labeled with specific subsets (13). A randomized controlled study of healthy elderly adults (55 to 70 years old) in Japan discovered that the plasma phospholipid content of AA increased dose-dependently following 4 weeks of AA supplementation at 0.24 g/day or 0.73 g/day. However, this supplementation did not influence the levels of AA metabolites and the plasma concentrations of CRP, IL-6, and TNF-α (14). The provided evidence indicates that increasing AA intake results in elevated AA content in plasma or cellular phospholipids. However, it does not exert a notable impact on pro-inflammatory cytokine production and the number of inflammatory cells. In parallel, studies focusing on LA, a synthetic substrate of AA, revealed that increasing LA intake did not elevate AA concentrations in plasma or PBMC. Moreover, it was not significantly associated with multiple inflammatory markers (39–41). This observation could be attributed to the saturation of the pathway for synthesizing AA from LA. Conversely, some studies even suggest that AA and LA may be associated with reduced inflammation (15–17). A cross-sectional study of 364 patients with CVD secondary prevention showed that augmented dietary consumption of ω-3 and ω-6 PUFAs was inversely correlated with levels of CRP, IL-1β, IL-10, and IL-12 (16). Another cross-sectional study showed that ω-3 and ω-6 PUFAs intake was linked to a reduced risk of developing CVD in comparison with intake of either fatty acid alone (17). These findings align with our observation that ω-6 PUFA intake is inversely associated with inflammatory markers. The inhibitory effect of ω-6 PUFAs on inflammatory responses may be achieved through eicosanoid-independent pathways as well as the production of precursors related to the inflammatory abrogation mediators. Nonetheless, additional research is required to explore the specific mechanisms involved (42).

In addition, this study revealed no noteworthy correlation between ω-6: ω-3 ratio and SII, along with other secondary measures. It is noteworthy that research investigating the relationship between the ω-6: ω-3 ratio and inflammatory markers has yielded conflicting results (18–22). Kalogeropoulos et al. (18) demonstrated a strong relationship between the proportion of ω-6: ω-3 and hs-CRP, IL-6, TNF-α, fibrinogen, and homocysteine in serum from 374 healthy people in the ATTICA’s study database. This suggests that the inflammatory balance of the body may be regulated by the relative amounts of ω-6 and ω-3 fatty acids. Another study involving 1,123 healthy individuals discovered an inverse relationship between the proportion of ω-6: ω-3 in fasting plasma and the anti-inflammatory marker IL-10 (20). Zhang et al. (19) utilized a population cohort from the UK Biobank to identify an elevated risk of all-cause, cancer, and CVD mortality with an elevated ω-6: ω-3 ratio in the population. Harris (21, 22) conducted an analysis of 11 case–control and two prospective cohort studies, revealing that the ω-6: ω-3 ratio was not effective in distinguishing coronary artery disease cases from healthy subjects. Therefore, it is suggested that more evidence is necessary to substantiate the utility of the ω-6: ω-3 ratio as a biomarker for predicting disease status or serving as a nutritional reference. This is supported by our findings. The ω-6: ω-3 ratio may not allow for the efficacy of each fatty acid to be assessed individually. Therefore, no recommendation can be given for a more accurate assessment.

The specific mechanism for the “L” shaped dose–response relationship between dietary ω-3 and ω-6 fatty acid intake and SII index is unclear, but there are several possibilities. First, dietary fatty acid intake is strongly associated with age, BMI, and individual metabolism. The results of stratified analyses show that there are some differences in BMI among the included study populations. One study found that a high dietary ω-6: ω-3 PUFA ratio was positively associated with excessive obesity and worsened metabolic status (43). And BMI is a better predictor of response to dietary supplements than simple body weight (44). Therefore, BMI differences in the study population may contribute to the over-activation of the inflammatory state, resulting in an “L” shaped dose–response relationship between PUFAs intake and inflammatory biomarkers in the body. Secondly, the effect of a single dietary component on homeostasis is limited, as the structure and function of cell membranes are regulated by other dietary factors, such as antioxidants and polyphenols, in addition to PUFAs. Several studies have shown that PUFAs is highly susceptible to oxidation, and their peroxidation produces lipid peroxides, which can harm the organism. The dietary intake of PUFA is accompanied by the intake of certain antioxidants, such as LA and vitamin E which are also obtained through vegetable oils. An epidemiological study investigating the relationship between PUFA intake and CRP concentrations found that the negative correlation between dietary ω-3 and ω-6 fatty acid intake and elevated CRP was only significant in individuals with low vitamin E intake, demonstrating some interaction between vitamin E and PUFAs (45). Thus with the gradual increase in ω-3 and ω-6 fatty acid intake, antioxidants in the body are unable to antagonize the higher levels of lipid peroxides and an increase follows a decrease in the level of inflammation. In addition to this, PUFA is also closely associated with the platelet-activating factor (PAF), the synthesis and catabolism of which involves the participation of a series of enzymes, among which lipoprotein-associated phospholipase A2 (Lp-PLA2) is considered to be a marker of vascular inflammatory response in the body. A cross-sectional study including 2,246 participants found that AA, EPA, and DHA plasma levels were negatively correlated with Lp-PLA2 mass and activity (46). In addition, a negative correlation between EPA and DHA and Lp-PLA2 concentrations was also observed in adipose tissue (47). Further studies revealed that this may affect Lp-PLA2 expression through activation of p38 mitogen-activated protein kinase and phosphatidylinositol 3-kinase (48–50). This may be related to the “L” shaped relationship between ω-3 and ω-6 fatty acid intake and SII index. However, the association between ω-3 and ω-6 PUFAs and PAF has not yet been established, and further studies are needed (51, 52).

On the one hand, this research exhibits multiple notable strengths. Firstly, it examined the correlation between dietary ω-3, ω-6 PUFAs intake and ω-6: ω-3 ratio and systemic immune-inflammatory status, utilizing multiple inflammatory indicators. Secondly, it explored the dose–response relationship between the main indicator SII and the dietary intake of ω-3 and ω-6 PUFAs. Finally, it employed a large, nationally representative sample, which enhances statistical power and augments the accuracy and reliability of the findings. On the other hand, this research also has certain limitations. Firstly, due to its cross-sectional design, establishing causality is challenging. Secondly, dietary data were collected via a 24-h dietary recall interview, which may lead to inaccuracy.

## 5 Conclusion

This research proposes that there is an inverse relationship between ω-3, ω-6 PUFAs intake and systemic inflammatory biomarkers in humans. The intake ω-3 and ω-6 PUFAs in the dose–response relationship exhibited an “L”-type association with the SII, indicating an initial decrease followed by an increase. However, no noticeable association was observed between ω-6: ω-3 ratio and these inflammatory markers. Further investigation is warranted to elucidate the mechanisms underlying this observation.

## Data availability statement

The original contributions presented in the study are included in the article/Supplementary material, further inquiries can be directed to the corresponding author.

## Ethics statement

The studies involving humans were approved by National Center for Health Statistics. The studies were conducted in accordance with the local legislation and institutional requirements. The participants provided their written informed consent to participate in this study. Written informed consent was obtained from the individual(s) for the publication of any potentially identifiable images or data included in this article.

## Author contributions

YL: Data curation, Formal analysis, Methodology, Software, Validation, Writing – original draft, Writing – review & editing. HT: Conceptualization, Data curation, Formal analysis, Methodology, Writing – original draft. XY: Conceptualization, Methodology, Visualization, Writing – original draft. LilM: Formal analysis, Software, Visualization, Writing – review & editing. HZ: Data curation, Supervision, Visualization, Writing – review & editing. GZ: Data curation, Formal analysis, Methodology, Software, Writing – review & editing. XC: Conceptualization, Data curation, Formal analysis, Writing – original draft. LijM: Data curation, Methodology, Writing – original draft. JG: Formal analysis, Software, Validation, Writing – review & editing. WJ: Conceptualization, Methodology, Visualization, Writing – review & editing.
