# Supplementary material for: Associations of ω-3, ω-6 polyunsaturated fatty acids intake and ω-6: ω-3 ratio with systemic immune and inflammatory biomarkers: NHANES 1999-2020
Source: Front Nutr. 2024 Jun 7;11:1410154. doi: 10.3389/fnut.2024.1410154 (PMC11190316; doi:10.3389/fnut.2024.1410154)
Supplement: Supplementary file 1 [file Table_1.docx]

Supplementary Material

Associations of ω-3, ω-6 Polyunsaturated Fatty Acids Intake and ω-6: ω-3 Ratio with systemic immune and inflammatory biomarkers: NHANES 1999-2020

Yifan Li^1†^, Hao Tang^1,2†^, Xiaotong Yang^1^, Lili Ma^1^, Hangqi Zhou^1^, Guangjiang Zhang^1^, Xin Chen^1^, Lijun Ma^1^, Jing Gao^1^ and Wei Ji ^3^*

*** Correspondence:** Wei Ji:[weiweiji1103@163.com](mailto:weiweiji1103@163.com)

# Supplementary Tables

Supplementary Table 1. Stratified analyses of the associations between dietary ω-3 PUFAs intake and SII.

| **variable** | **Total ω-3 PUFAs intake(g)** |  |  |
| --- | --- | --- | --- |
| **SII** | **β（95%CI）*p-*Value** | ***p*** | ***p* for interaction** |
| **Sex** |  |  | 0.11 |
| Male | -2.334 (-7.647,2.979) | 0.387 |  |
| Female | -8.649 (-14.538,-2.759) | 0.004 |  |
| **Age** |  |  | 0.373 |
| 20-40 | -7.317 (-13.276,-1.358) | 0.016 |  |
| 41-60 | -5.624 (-11.764,0.515) | 0.072 |  |
| >=60 | -12.977 (-22.228,-3.727) | 0.006 |  |
| **Smoke** |  |  | 0.523 |
| never | -7.429 (-12.521,-2.338) | 0.004 |  |
| former | -11.709 (-19.784,-3.634) | 0.005 |  |
| current | -4.71 (-14.870,5.449) | 0.361 |  |
| **Race** |  |  | 0.841 |
| Mexican American | -12.583 (-23.499,-1.667) | 0.024 |  |
| Non-Hispanic White | -8.173 (-13.188,-3.159) | 0.002 |  |
| Non-Hispanic Black | -7.39 (-14.217,-0.564) | 0.034 |  |
| Other Hispanic | -14.091 (-26.874,-1.308) | 0.031 |  |
| Other race | -8.989 (-21.436,3.459) | 0.156 |  |
| **Body mass index** |  |  | 0.663 |
| <25 | -6.283 (-13.230,0.664) | 0.076 |  |
| 25-30 | -8.412 (-14.899,-1.925) | 0.011 |  |
| >30 | -10.492 (-16.946,-4.038) | 0.002 |  |
| **Education level** |  |  | 0.144 |
| Below high school | -22.214 (-37.267,-7.160) | 0.004 |  |
| High school | -10.427 (-18.601,-2.254) | 0.013 |  |
| Above high school | -5.606 (-9.883,-1.328) | 0.011 |  |

SII, systemic immune-inflammation index;β, standardized coefficients; CI, confidence interval.

Supplementary Table 2. Stratified analyses of the associations between dietary ω-6 PUFAs intake and SII.

| **variable** | **Total ω-6 PUFAs intake(g)** |  |  |
| --- | --- | --- | --- |
| **SII** | **β（95%CI）*p-*Value** | ***p*** | ***p* for interaction** |
| **Sex** |  |  | 0.884 |
| Male | -0.619 (-1.237,-0.002) | 0.049 |  |
| Female | -0.548 (-1.343,0.247) | 0.175 |  |
| **Age** |  |  | 0.39 |
| 20-40 | -1.047 (-1.792,-0.301) | 0.006 |  |
| 41-60 | -0.628 (-1.431,0.175) | 0.124 |  |
| >=60 | -1.561 (-2.648,-0.475) | 0.005 |  |
| **Smoke** |  |  | 0.58 |
| never | -1.097 (-1.735,-0.458) | <0.001 |  |
| former | -1.47 (-2.441,-0.500) | 0.003 |  |
| current | -0.719 (-1.768,0.330) | 0.178 |  |
| **Race** |  |  | 0.75 |
| Mexican American | -1.559 (-2.846,-0.271) | 0.018 |  |
| Non-Hispanic White | -1.221 (-1.826,-0.616) | <0.0001 |  |
| Non-Hispanic Black | -0.814 (-1.668,0.040) | 0.062 |  |
| Other Hispanic | -1.155 (-2.686,0.375) | 0.138 |  |
| Other race | -0.323 (-2.074,1.428) | 0.716 |  |
| **Body mass index** |  |  | 0.881 |
| <25 | -0.997 (-1.903,-0.091) | 0.031 |  |
| 25-30 | -1.231 (-1.991,-0.470) | 0.002 |  |
| >30 | -1.272 (-2.037,-0.506) | 0.001 |  |
| **Education level** |  |  | 0.316 |
| Below high school | -2.334 (-4.261,-0.408) | 0.018 |  |
| High school | -1.314 (-2.199,-0.429) | 0.004 |  |
| Above high school | -0.842 (-1.405,-0.279) | 0.004 |  |

SII, systemic immune-inflammation index;β, standardized coefficients; CI, confidence interval.
